# Increasing Rate of Mortality Due to HIV/AIDS in Iranian Children: An Alarm for Health Policymakers

**Published:** 2017-07

**Authors:** Salman KHAZAEI, Shahab REZAEIAN

**Affiliations:** 1. Dept. of Epidemiology, School of Public Health, Hamadan University of Medical Sciences, Hamadan, Iran; 2. Research Center for Environmental Determinants of Health (RCEDH), Kermanshah University of Medical Sciences, Kermanshah, Iran

## Dear Editor-in-Chief

In the recent decade, substantial reductions in child mortality rate occurred in both developed and developing countries. However, these achievements are now being threatened in many developing countries by the emerging of the HIV/AIDS epidemic, such that the decline in trend of child mortality rates has decreased in some countries and begun to reverse direction in others (1).

In Iran, more than 50% of HIV cases occurred in 25–34 yr old as an active age group and in the age of marriage. Therefore, HIV/AIDS can directly transmit through vertical transmission and through orphanhood and inadequate care for newborn indirectly affect child death (2).

**Fig. 1:**
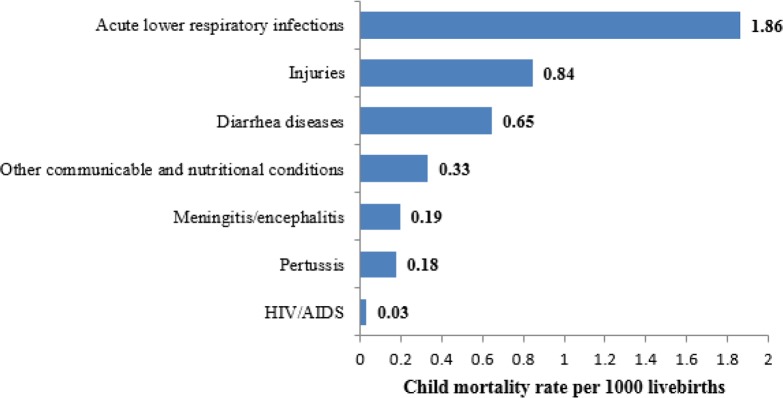
Mortality rates (per 1000 live births) among Iranian children under 5 yr (1–59 month), by cause of death in 2015

Although HIV is not among the leading cause of child death in Iran and only in 2015, nearly 0.03 per l000 live births in children were attributed to HIV/AIDS (Fig. 1), but the worrying issue is an increasing trend in the rate of deaths from AIDS during 2000–2015.

**Fig. 2:**
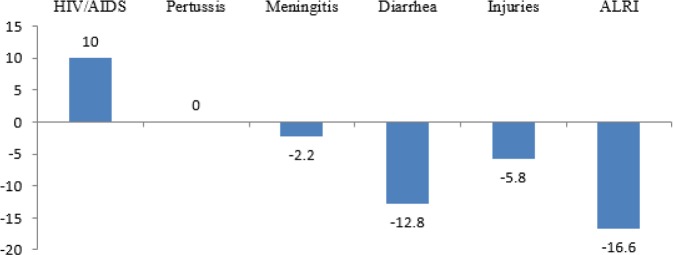
Average annual rate of reduction/increase for some of child deaths in Iran

There was a significant decreasing trend of mortality rates among children for diarrhea diseases (−12.8% annually), injuries (−5.8% annually), acute lower respiratory infections (−16.6% annually) and meningitis/encephalitis (−2.2% annually). However, the trend of HIV/AIDS mortality rate among children under 5 yr increased from 0.0078 in 2000 to 0.021 per 1000 live births in 2015 (+10% annually) (Fig. 2) (3).

The majority of infections among children living with HIV occur through mother-to-child transmission. Although it is vital to receive antiretroviral treatment (ART) to keep healthy, more of pregnant women and HIV-exposed infants are not received ART. Accordingly, without ART, a third of HIV-infected children will not reach their first birthday, and half will not reach their second birthday (4).

On the other hand, considering the high lifetime costs of care for HIV-infected children, which can avert by screening pregnant women for HIV and leads to gain in life years for both mothers and children? Moreover, the higher odds of HIV-testing among pregnant women (5) lead to early detection of HIV infection and can reduce the risk of mother to child transmission (6).

New infections and upward trend of HIV/AIDS in children will remain unless these mentioned factors above with funding, trained staff and resources are addressed.
